# Transition to targeted transperineal prostate biopsies does not impact progression to curative treatment for patients initially entered into active surveillance

**DOI:** 10.1177/03915603251395510

**Published:** 2025-11-23

**Authors:** Alfred Honore, Christian Arvei Moen, Lars Anders Rokne Reisæter, Karsten Gravdal, Patrick Juliebø-Jones, Christian Beisland

**Affiliations:** 1 Department of Urology, Haukeland University Hospital, Bergen, Norway; 2 Department of Clinical Medicine, University of Bergen, Norway; 3 Department of Radiology, Haukeland University Hospital, Bergen, Norway; 4 Department of Pathology, Haukeland University Hospital, Bergen, Norway

**Keywords:** transperineal, prostate biopsy, targeted, active surveillance, outcomes, safety, LATP, cognitive, fusion, biopsy

## Abstract

**Background:**

Concerns over infection have driven a shift from transrectal to transperineal prostate biopsy, while pre-biopsy MRI has promoted a move from systematic to targeted sampling. These changes may impact patient selection, treatment planning, and risk stratification in active surveillance.

**Objective:**

To compare active surveillance outcomes of patients diagnosed primarily by targeted transperineal (tTP) biopsy versus standard transrectal (sTR) biopsy.

**Design, setting and participants:**

Prospectively collected data of men who underwent prostate biopsy between January 2018 and May 2022 who were included into active surveillance in our institution.

**Outcome measurements and statistical analysis:**

Comparison of patient characteristics, clinical and radiological features, positive and total number of biopsies, biopsy Gleason grade group (GG) at inclusion using simple descriptive statistics, groups compared using Wilcoxon rank sum test; Fisher’s exact test; Pearson’s Chi-squared test. Time to transition to curative treatment was calculated using the Kaplan-Meier plot.

**Results:**

There were 112 and 167 patients in the tTP and sTR groups, respectively. No significant differences in age, BMI, ECOG, Charlson Comorbidity Index, PSA, radiological T-stage or GG at inclusion was seen. Number of positive biopsy cores were unchanged between tTP and sTR at 2 (1–3) (median (IQR); *p* = 0.2), while total cores were reduced significantly to 3 (3–5) from 12 (8–12) (*p* < 0.001), respectively. Overall, there was no difference in progression from surveillance to active treatment (*p* = 0.084), but when separated by biopsy type and GG, there was a significantly higher rate of transitioning to curative treatment after 1 year in the sTR group with GG2+ at inclusion (*p* < 0.0001), compared to the other three.

**Conclusion:**

Using targeted transperineal biopsy of the index lesion(s) alone does not lead to increased treatment of patients included in active surveillance.

## Introduction

Active surveillance (AS) of prostate cancer (PCa) was first conceived of by Klotz. The first reports of its application in a clinical setting were published in 2010, and long-term follow-up results have since confirmed its safety as an approach for low-risk disease.^1^ This treatment strategy was devised based on the understanding that many patients with low grade adenocarcinomas die of other causes. Furthermore, findings from long term follow-up studies have shown that in many cases, the disease was indolent.^2,3^ The ProtecT trial has also shown the clinical safety of a deferred treatment approach in the setting of low risk PCa.^4^ AS is therefore a recommended treatment strategy for this disease group.^5^ With the introduction of magnetic resonance imaging (MR/MRI) to the diagnostic pathway, there has also been a subsequent effect on AS protocols. One of the main arguments for the MRI and targeted biopsy pathway is to avoid diagnosing low grade tumours and thus obviate the need for unnecessarily burdening both patients and the health care system.^6^ This, in turn, has led to the increased inclusion of Gleason Grade Group (GG) 2 (and higher) tumours. This is associated with a higher rate of unfavourable pathology at operative treatment.^7^ Recently, there has been an increase in the use of the transperineal method due to lower rates of post biopsy infection.^8–10^ It is also the preferred route according to the current European Association of Urology (EAU) guidelines.^5^ At our institution, as in most centres worldwide, the standard of care was transrectal prostate biopsy performed under antibiotic prophylaxis. However, we observed a high rate of post-procedural infections with this technique. Consequently, in 2020 we began a gradual transition to cognitive freehand transperineal prostate biopsy under local anaesthesia (LATP), adopting this method exclusively by 2022. Among patients undergoing LATP, we observed a marked reduction in infectious complications, despite antibiotic prophylaxis being given to only 2.4% of patients.^11^ We therefore aimed to compare active surveillance (AS) inclusion and outcomes in patients diagnosed using the newer targeted transperineal (tTP) biopsy method with those diagnosed using the previous standard, systematic transrectal (sTR) biopsy.

## Materials and methods

### Patient cohorts

**Figure 1.**
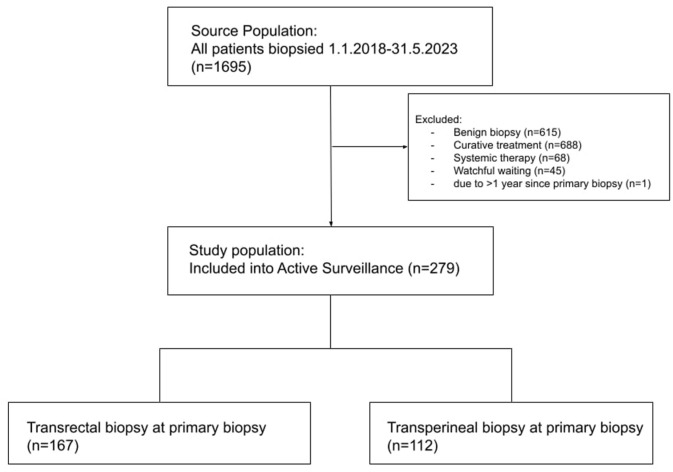
Inclusion diagram. Flowchart of patient inclusion into active surveillance.

The study was approved by the regional ethics committee (REK 2022-465105). Patients were eligible if they underwent a confirmatory biopsy for active surveillance (AS) at Haukeland University Hospital (HUH), a tertiary centre in Western Norway, between January 1st, 2018, and May 31st, 2022. This yielded 279 patients. For some, the initial (primary) biopsy had been performed before 2018. Ethical approval allowed us to retrospectively review their medical records to obtain primary biopsy data. Thus, all patients included had complete data on both primary and confirmatory biopsies. Cohorts were categorised by primary biopsy access route and type, and intention-to-treat (ITT) analyses were based on the conclusions of the multidisciplinary team meeting (MDT) at inclusion and follow-up. The inclusion process is shown in Figure 1. Recommendations for conversion to curative treatment at MDT review were guided by predefined criteria, including upgrading of GG, increased number of positive biopsy cores, greater cancer length per core, higher proportion of Gleason pattern 4, and/or progression on MRI. Of note, final decisions were made on an individual basis, with the MDT integrating these findings alongside each patient’s overall clinical context. This recommendation was discussed with the patient’s personal preference as part of shared decision making. Patients who preferred AS despite an MDT recommendation of curative treatment and who were later re-biopsied, were also included. Patients who were primarily recommended AS but opted for curative treatment, were excluded.

### Patient evaluation

Patient characteristics, biopsy GG, clinical and radiological T- stage, positive and total number of biopsy-cores were all recorded. Biopsy results were described using the International Standard of Urological Pathology (ISUP) grading system from 2019.^12^ MRI scans were performed using a 1.5- or 3-T scanner without an endorectal probe and reported by the same radiologist according to the PIRADs v2 and later PIRADs 2.1 systems.^13,14^ Mainly three biopsies were taken per visible PIRADs ⩾ 3 lesion identified on MRI in the tTP group. However, if PSA-density (PSAD) was <0.20 and changes were diffuse, biopsies were omitted. The default approach in the sTR group was to take 12 biopsies, with the option of targeted biopsy by sector. If there were more than six biopsies taken in one patient of either group, it was not considered purely targeted and thus considered standard, despite the possibility for a combination of the two. Progression was deemed at the MDT based on the repeat MRI and surveillance biopsy findings. If biopsies were benign or unchanged, continued AS was recommended but with specific instructions of re-referral if PSAD increased to >0.20, or a >50% rise if PSAD was >0.20 already.

### Data synthesis and analysis

Data was collected prospectively using structured data forms in the hospital journal system. Patient cohorts were categorised by primary biopsy access technique (either transrectally or transperineally) and subdivided into GG1 and GG2 or higher (GG2+).

Continuous variables were reported using median with interquartile range (IQR), and categorical variables were reported using frequencies and proportions. All analyses and graph plots were done using the R4.2.2 build (R Foundation for Statistical Computing, Vienna, Austria). The Wilcoxon rank-sum test, Kruskal-Wallis rank sum test and Pearson’s χ^2^-test were used to compare groups. Tests were considered significant for *p* < 0.05. Progression to curative treatment was evaluated by the Kaplan-Meier plot. Converting to watchful waiting was not considered as an event.

## Results

### Patient cohort

The overall biopsy findings and ITT of the source population are summarised in Table 1. The clinical characteristics of the AS cohort of 279 patients are summarised in Table 2. There were no significant differences in age, BMI, ECOG, Charlson Comorbidity Index or PSA. There was an overall reduction in benign biopsies and patients entering AS, and an increase in primary curative treatment in the tTP group.

**Table 1.** Overall intention to treat.

Characteristic	Transperineal, *N* = 832^a^	Transrectal, *N* = 863^a^	*p*-value^b^
Treatment-intention			<0.001
Active surveillance	112 (13%)	167 (19%)	
Benign	273 (33%)	342 (40%)	
Curative treatment	384 (46%)	304 (35%)	
Systemic therapy	31 (3.7%)	37 (4.3%)	
Watchful waiting	32 (3.8%)	13 (1.5%)	

Active surveillance: surveillance as per hospital protocol and national guidelines; Benign biopsy (no treatment); curative treatment: radiation therapy or radical prostatectomy; systemic therapy: treatment for high burden metastatic disease. watchful waiting: passive observation until signs of symptomatic progression.

a *n*(%).

b Pearson’s Chi-squared test.

**Table 2.** Active surveillance patient characteristics.

Characteristics	Transperineal, *N* = 112^a^	Transrectal, *N* = 167^a^	*p*-value^b^
Age at inclusion	66 (62, 71)	66 (62, 69)	0.6
BMI	26.0 (24.3, 28.4)	25.5 (23.7, 27.8)	0.5
ECOG			> 0.9
0	109 (97%)	162 (97%)	
1+	3 (2.7%)	5 (3.0%)	
ASA			0.10
1–2	102 (91%)	160 (96%)	
3+	10 (8.9%)	7 (4.2%)	
Charlson Comorbidity Index (age adjusted)			0.7
0	4 (3.6%)	5 (3.0%)	
1	15 (13%)	26 (16%)	
2	44 (39%)	72 (43%)	
3	35 (31%)	40 (24%)	
4+	14 (13%)	24 (14%)	
PSA	6.8 (5.7, 9.0)	7.1 (5.4, 9.3)	0.7
Prostate volume	36 (29, 49)	43 (31, 57)	0.014
PSA density	0.18 (0.14, 0.27)	0.16 (0.12, 0.24)	0.029
Clinical T-stage			0.018
1	93 (83%)	118 (71%)	
2	19 (17%)	49 (29%)	
Radiological T-stage			0.4
T1c/T2a	67 (60%)	105 (65%)	
T2b/c	42 (38%)	49 (30%)	
T3	3 (2.7%)	7 (4.3%)	
PIRAD (v2.0/2.1)			0.032
2	0 (0%)	5 (3.1%)	
3	31 (28%)	63 (39%)	
4	62 (55%)	75 (47%)	
5	19 (17%)	18 (11%)	
Positive cores	2 (1, 3)	2 (1, 3)	0.2
Total cores	3 (3, 5)	12 (8, 12)	< 0.001
ISUP GG			0.2
1	82 (73%)	134 (80%)	
2+	30 (27%)	33 (20%)	

BMI: body mass index; ECOG: Eastern Conglomerate Oncology Group performance status; ASA: American Society of Anaesthesiology grade; PSA: prostate specific antigen; PIRAD: prostate imaging reporting and data system; ISUP: International Society of Uropathologists; GG: Gleason grade group.

a Median (IQR); *n* (%).

b Wilcoxon rank sum test; Fisher’s exact test; Pearson’s Chi-squared test.

### Patient characteristics

Patient characteristics are summarised in Table 2. There was no difference in radiological T-stage or ISUP grade at inclusion in the AS cohorts. There were, however, significantly fewer PIRAD 3 lesions in the tTP versus sTR group (28% vs 39%), while there were more PIRAD 4 (55% vs 47%) and PIRAD 5 (17% vs 11%) lesions in the tTP group, and no PIRAD 2 lesions (*p* = 0.032). While there was no difference in PSA, PSAD was significantly higher in the tTP group (0.18 (0.14–0.27) vs 0.16 (0.12–0.24), (*p* = 0.029). On the other hand, there were more palpable cT2 tumours in the sTR group. There was no difference in positive biopsy cores of 2 (1–3) (*p* = 0.2) but a significant reduction of total cores from 12 (8–12) to 3 (3–5) (*p* < 0.001) between the sTR and tTP groups, respectively.

### Comparison of outcomes

**Figure 2.**
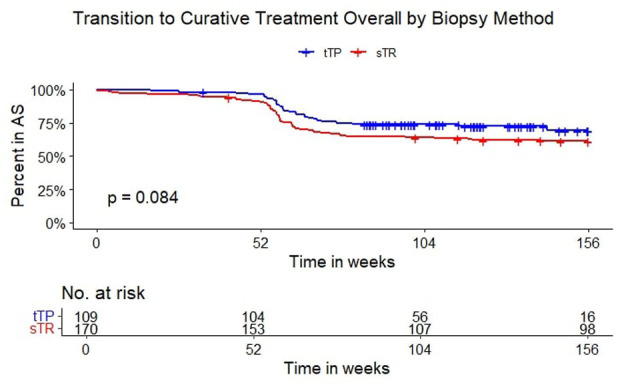
Transition to curative treatment overall by access. AS: active surveillance; blue line: targeted transperineal biopsy (tTP); red line: standard transrectal biopsy (sTR); inset: number at risk.

**Figure 3.**
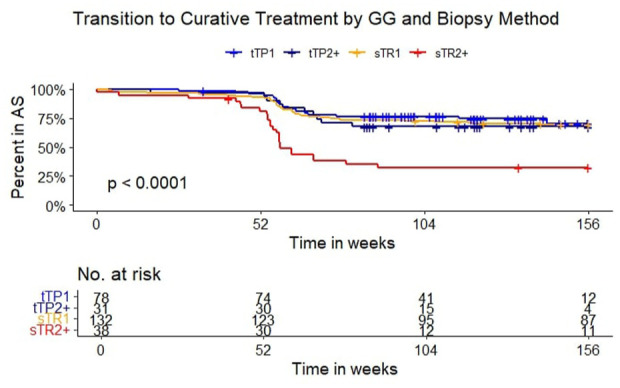
Transition to curative treatment by grade group and access. AS: active surveillance; blue line: targeted transperineal biopsy and grade group 1 (tTP1); purple line: targeted transperineal biopsy and grade group 2 or more (tTP2+); yellow line: standard transrectal biopsy and grade group 1 (sTR1); red line: standard transrectal biopsy and grade group 2 or more (sTR2+): GG – ISUP grade group; Inset: number at risk.

There were overall fewer conversions from AS to curative treatment in the tTP group compared to the sTR group, but this was not statistically significant (*p* = 0.084; Figure 2). When separating by biopsy type and GG, there was a significantly higher rate of conversion to curative treatment after 1 year in the sTR group with GG2+ at inclusion, compared to the other three (Figure 3 and Table 3, *p* < 0.0001). The last biopsy in the transperineal group was targeted in 95% and 94% for those with GG1 and GG2 at primary biopsy, respectively. In the transrectal group, the last biopsy was targeted in 80% and 62% of those with GG1 and GG2+ at primary biopsy, respectively. This coincided with a 65% conversion to active treatment in the GG2+ category of the sTR group. The median follow-up time in the sTR/GG2+ group was also shorter than the tTP/GG2+ group and had the same number of patients. The sTR/GG1 group had the longest median follow up time of 212 months, with a 41% conversion to active treatment where GG progression was seen in 46%. The median time was only 108 weeks in the tTP group, with a 38% GG progression rate. The most common last biopsy method was targeted, irrespective of access route. One patient was registered as having their last biopsy as a TURP but this didn’t affect conversion to active treatment as he entered watchful waiting and was censored at that date.

**Table 3.** Outcomes by primary access and grade group.

Characteristic	Transperineal and GG1, *N* = 78^a^	Transperineal and GG2+, *N* = 31^b^	Transrectal and GG1, *N* = 130^a^	Transrectal and GG2+, *N* = 34^a^	*p*-value^b^
Conversion to curative treatment	21 (27%)	10 (32%)	53 (41%)	22 (65%)	0.002
GG progression at biopsy	29 (38%)	5 (17%)	56 (46%)	8 (24%)	0.008
Time to progression	108 (85, 141)	93 (71, 127)	212 (75, 253)	58 (54, 183)	<0.001
Initial biopsy type					<0.001
Standard	10 (13%)	5 (16%)	103 (79%)	22 (65%)	
Targeted	68 (87%)	26 (84%)	27 (21%)	12 (35%)	
Last biopsy type					<0.001
Standard	4 (5.1%)	2 (6.5%)	26 (20%)	13 (38%)	
Targeted	74 (95%)	29 (94%)	102 (80%)	21 (62%)	
Last biopsy access route					<0.001
TURP	0 (0%)	0 (0%)	1 (0.9%)	0 (0%)	
Transperineal	75 (99%)	30 (100%)	71 (61%)	10 (29%)	
Transrectal	1 (1.3%)	0 (0%)	45 (38%)	24 (71%)	

GG: Gleason grade group; TURP: trans urethral resection of the prostate.

a *n* (%); Median (IQR).

b Pearson’s Chi-squared test; Kruskal-Wallis rank sum test; Fisher’s exact test.

## Discussion

### Summary of findings

In this study, we found that there were fewer patients included into AS in the tTP group due to a decrease in patients with GG1 disease, but with a similar number of patients with GG2+. The number of positive biopsies at primary biopsy were the same in both cohorts, despite significantly more biopsies taken in the sTR group. There were also more targeted biopsies performed at surveillance biopsy in both cohorts, reflecting a change in practice over time. There was a much higher rate of conversion to active treatment in the sTR patients with primary GG2+, where there was also a shorter median follow-up time compared to the tTP group which was introduced later.

### Reduction of AS by increased targeted biopsy

The rationale for adopting MRI selection and targeted biopsies is to avoid the over diagnosis of low risk PCa, which is in keeping with the findings of the PRECISION study.^6^ We saw a proportional increase in PIRAD 4 and 5 lesions, but this was due to a reduction of PIRAD 3 and 2 lesions, and fewer patients with GG1 findings on pathology. Giganti et al. showed that patients with lesions not visible on MRI developed these after a mean time of 3.6 years, reflecting a true progression.^15^ In other words, a considerable lead time bias that should favour postponing or even omitting biopsy in general if there are no visible lesions. The inclusion of intermediate risk PCa, the use of MRI for detection and follow-up, and an increase in targeted biopsies is also well established.^16–18^ However, the use of targeted biopsies is most often done by software fusion.^19^ We have noted a similar trend with our transition, with an increase in conversion to curative treatment due to the increased use of cognitively targeted biopsies, irrespective of access route. There is a concern of grade migration and thus overtreatment due to increased sampling of the tumour.^20^ A systematic review by Weinstein et al. found that more cancers were downgraded at final pathology when systematic and targeted biopsies were combined, compared to systematic biopsies alone.^21^ We however found that the cognitively targeted transperineal MRI fusion pathway results in a significantly higher rate of GG4/5 cancers at final pathology but this was due to patient selection and not a change in concordance.^22^ Another multicentre study by Baboudjian et al. found no evidence that targeted biopsy leads to overtreatment.^23^ There was also a trend to more upgrading and less downgrading in the tTP group, although this was not significant. It is possible that cognitive transperineal fusion biopsies are slightly less precise than software fusion (often using a jig or rigid robotic arm), giving an effect similar to perilesional biopsies that have been shown to increase precision of sampling.^24,25^ We would therefore argue that by showing restraint in the number of biopsies we take, over-sampling and over-grading is less likely. By doing LATP using a cognitive approach, which is low cost and without the need for expensive fusion equipment or an operating room, it is easily distributable to office urology. We have also found it to be reproducible between operators and thereby a safe practice.^11^

### Positive biopsies and disease severity

Despite a radical reduction in the total number total biopsies, the overall number of positive biopsies were the same in both cohorts. We have previously shown that cognitive biopsy is reproducible between operators and therefore assume operator independence.^11^ We therefore assume that patients included into AS, either by targeted or systematic biopsies, have low volume disease. Coincidentally, this is the number of positive cores that is recommended as the threshold for inclusion in AS, and a previous meta-analysis found that > 2 cores and a high PSAD (as well as African descent) were the only factors predicting progression.^26^

### Progression and conversion to active treatment

Based on a higher rate of conversion to active treatment in the sTR/GG2+ subgroup by targeted biopsy as seen in Figure 3, we can assume that targeted biopsies result in fewer surprises at the first surveillance biopsy. It is more likely that the conversion rate is due to sampling error at the primary biopsy as opposed to a true cancer conversion, since the sTR/GG2+ and tTP/GG2+ subgroups were almost equal in size, and a paradoxical shorter median follow-up time in the sTR/GG2+. This is also supported by the finding of more targeted biopsies used at surveillance biopsy also in the sTR group. When comparing to the conversion rates in the ProtecT trial, there was a similar sharp initial rise within the first year.^4^ In that study, 13 patients died of PCa following prostatectomy where 46% had stage progression and 77% had grade progression. This could probably have been avoided by better precision at the primary workup. Because of this, we also noted fewer patients converting to curative treatment in both subcategories of the tTP arm, suggesting that this is a safer option for patients both in the primary and surveillance biopsy setting as it is better at selecting patients who likely require treatment.^27^ Moreover, there is no clear evidence that cognitive targeting is inferior to software fusion.^28^ This should be considered in future AS protocols, as the likelihood of unfavourable pathology at prostatectomy increases with core length and > 5% Gleason pattern 4 on biopsy.^7^

### Limitations

The main limitations of this study are the relatively short follow-up period, its single centre nonrandomized design, and the modest cohort sizes. In addition, changes over the study period in both access route and the increasing use of targeted biopsy in the primary setting introduce heterogeneity and potential bias. We also have no record of other possible treatments received in either group that may or may not have affected progression risk.^29^ A key strength of this study is that it demonstrates the successful implementation of a staged, and ultimately complete, transition from transrectal to transperineal biopsy, with increasing adoption on a targeted-only approach. This transition has reduced the total number of biopsy cores required while maintaining the integrity of patient selection for treatment.

## Conclusion

Using cognitive transperineal targeted biopsy of the index lesion(s) alone does not adversely affect outcomes of AS compared to standard transrectal biopsy and is a safe alternative to systematic biopsies. Targeted biopsies did indeed cause a greater conversion to active treatment, but only when the primary biopsy modality was systematic/standard and was not affected by access route.

## References

[1] Klotz L. Active surveillance for low-risk prostate cancer. Curr Opin Urol 2017; 27: 225–230. 28267056 10.1097/MOU.0000000000000393

[2] Sakr WA Grignon DJ Crissman JD , et al. High grade prostatic intraepithelial neoplasia (HGPIN) and prostatic adenocarcinoma between the ages of 20-69: an autopsy study of 249 cases. Vivo Athens Greece 1994; 8: 439–443. 7803731

[3] Popiolek M Rider JR Andrén O , et al. Natural history of early, localized prostate cancer: a final report from three decades of follow-up. Eur Urol 2013; 63: 428–435. 23084329 10.1016/j.eururo.2012.10.002

[4] Hamdy FC Donovan JL Lane JA , et al. Fifteen-year outcomes after monitoring, surgery, or radiotherapy for prostate cancer. N Engl J Med 2023; 388: 1547–1558. 36912538 10.1056/NEJMoa2214122

[5] Cornford P van den Bergh RCN Briers E , et al. EAU-EANM-ESTRO-ESUR-ISUP-SIOG guidelines on prostate cancer-2024 update. Part I: screening, diagnosis, and local treatment with curative intent. Eur Urol 2024; 86: 148–163. 38614820 10.1016/j.eururo.2024.03.027

[6] Kasivisvanathan V Rannikko AS Borghi M , et al. MRI-Targeted or standard biopsy for prostate-cancer diagnosis. N Engl J Med 2018; 378: 1767–1777. 29552975 10.1056/NEJMoa1801993 PMC9084630

[7] Björklund J Cheung DC Martin LJ , et al. Low-volume grade group 2 prostate cancer candidates for active surveillance: a radical prostatectomy retrospective analysis. Scand J Urol 2023; 57: 29–35. 36683418 10.1080/21681805.2023.2165709

[8] Gorin MA Meyer AR Zimmerman M , et al. Transperineal prostate biopsy with cognitive magnetic resonance imaging/biplanar ultrasound fusion: description of technique and early results. World J Urol 2020; 38: 1943–1949. 31679065 10.1007/s00345-019-02992-4

[9] Jacewicz M Günzel K Rud E , et al. Antibiotic prophylaxis versus no antibiotic prophylaxis in transperineal prostate biopsies (NORAPP): a randomised, open-label, non-inferiority trial. Lancet Infect Dis 2022; 22: 1465–1471. 35839791 10.1016/S1473-3099(22)00373-5

[10] Marra G Zhuang J Beltrami M , et al. Transperineal freehand multiparametric MRI fusion targeted biopsies under local anaesthesia for prostate cancer diagnosis: a multicentre prospective study of 1014 cases. BJU Int 2021; 127: 122–130. 32455504 10.1111/bju.15121

[11] Honoré A Moen CA Juliebø-Jones P , et al. Transitioning from transrectal to transperineal prostate biopsy using a freehand cognitive approach. BJU Int 2024; 133: 324–331. 38009392 10.1111/bju.16237

[12] van Leenders GJLH van der Kwast TH Grignon DJ , et al. The 2019 International Society of Urological Pathology (ISUP) consensus conference on grading of prostatic carcinoma. Am J Surg Pathol 2020; 44: e87–e99. 10.1097/PAS.0000000000001497 PMC7382533 32459716

[13] Weinreb JC Barentsz JO Choyke PL , et al. PI-RADS prostate imaging - reporting and data system: 2015, version 2. Eur Urol 2016; 69: 16–40. 26427566 10.1016/j.eururo.2015.08.052 PMC6467207

[14] Turkbey B Rosenkrantz AB Haider MA , et al. Prostate imaging reporting and data system version 2.1: 2019 update of prostate imaging reporting and data system version 2. Eur Urol 2019; 76: 340–351. 30898406 10.1016/j.eururo.2019.02.033

[15] Giganti F Moore CM Punwani S , et al. The natural history of prostate cancer on MRI: lessons from an active surveillance cohort. Prostate Cancer Prostatic Dis 2018; 21: 556–563. 30038388 10.1038/s41391-018-0058-5

[16] Loeb S Folkvaljon Y Bratt O , et al. Defining intermediate risk prostate cancer suitable for active surveillance. J Urol 2019; 201: 292–299. 30240688 10.1016/j.juro.2018.09.042

[17] Nassiri N Margolis DJ Natarajan S , et al. Targeted biopsy to detect Gleason score upgrading during active surveillance for men with low versus intermediate risk prostate cancer. J Urol 2017; 197: 632–639. 27639713 10.1016/j.juro.2016.09.070 PMC5315577

[18] Dall’Era MA Klotz L. Active surveillance for intermediate-risk prostate cancer. Prostate Cancer Prostatic Dis 2017; 20(1): 1–6. 27801900 10.1038/pcan.2016.51 PMC5303136

[19] Montorsi F Gandaglia G Briganti A , et al. Re: Veeru Kasivisvanathan, Armando Stabile, Joana B. Neves, et al. Magnetic resonance Imaging-targeted biopsy versus systematic biopsy in the detection of prstate cancer: a systematic review and meta-analysis. Eur Urol 2019;76:284–303. Eur Urol 2019; 76: e132. 31400947 10.1016/j.eururo.2019.07.042

[20] Vickers AJ . Effects of Magnetic Resonance Imaging Targeting on Overdiagnosis and Overtreatment of Prostate Cancer. Eur Urol 2021;80(5):567–72. 10.1016/j.eururo.2021.06.026 PMC8530856 34294510

[21] Weinstein IC Wu X Hill A , et al. Impact of magnetic resonance imaging targeting on pathologic upgrading and downgrading at prostatectomy: a systematic review and meta-analysis. Eur Urol Oncol 2023; 6: 355–365. 37236832 10.1016/j.euo.2023.04.004

[22] Honoré A Gravdal K Juliebø-Jones P , et al. Concordance with final pathology when transitioning from standard transrectal to cognitive targeted transperineal prostate biopsy. BJUI Compass 2025; 6: e486. 10.1002/bco2.486 PMC11771486 39877578

[23] Baboudjian M Diamand R Uleri A , et al. Does overgrading on targeted biopsy of magnetic resonance imaging-visible lesions in prostate cancer lead to overtreatment? Eur Urol 2024; 86: 232–237. 38494379 10.1016/j.eururo.2024.02.003

[24] Brisbane WG Priester AM Ballon J , et al. Targeted prostate biopsy: umbra, penumbra, and value of perilesional sampling. Eur Urol 2022; 82: 303–310. 35115177 10.1016/j.eururo.2022.01.008

[25] Noujeim J-P Belahsen Y Lefebvre Y , et al. Optimizing multiparametric magnetic resonance imaging-targeted biopsy and detection of clinically significant prostate cancer: the role of perilesional sampling. Prostate Cancer Prostatic Dis 2023; 26: 575–580. 36509930 10.1038/s41391-022-00620-8

[26] Petrelli F Vavassori I Cabiddu M , et al. Predictive factors for reclassification and relapse in prostate cancer eligible for active surveillance: a systematic review and meta-analysis. Urology 2016; 91: 136–142. 26896733 10.1016/j.urology.2016.01.034

[27] Santoro AA Di Gianfrancesco L Racioppi M , et al. Multiparametric magnetic resonance imaging of the prostate: lights and shadows. Urologia 2021; 88: 280–286. 34075837 10.1177/03915603211019982

[28] Watts KL Frechette L Muller B , et al. Systematic review and meta-analysis comparing cognitive vs. Image-guided fusion prostate biopsy for the detection of prostate cancer. Urol Oncol 2020; 38: 734.e19–734.e25. 10.1016/j.urolonc.2020.03.020 32321689

[29] Bizzarri FP Campetella M Ragonese M , et al. The role of alternative medicine and complimentary therapies in urologic disease: new horizons. Urologia 2024; 91: 641–646. 39045632 10.1177/03915603241258697

